# Enriching Nano‐Heterointerfaces in Proton Conducting TiO_2_‐SrTiO_3_@TiO_2_ Yolk–Shell Electrolyte for Low‐Temperature Solid Oxide Fuel Cells

**DOI:** 10.1002/advs.202401008

**Published:** 2024-06-12

**Authors:** Mengchen Du, Shaozheng Ji, Pan Zhang, Yongfu Tang, Yanyan Liu

**Affiliations:** ^1^ State Key Laboratory of Metastable Materials Science and Technology (MMST) Hebei Key Laboratory of Applied Chemistry Yanshan University Qinhuangdao 066004 P. R. China; ^2^ Ultrafast Electron Microscopy Laboratory School of Physics Nankai University Tianjin 300071 China

**Keywords:** interfacial conduction, LT‐SOFC nano‐heterointerfaces, proton conduction, yolk–shell heterostructure

## Abstract

A challenging task in solid oxide fuel cells (SOFCs) is seeking for an alternative electrolyte, enabling high ionic conduction at relatively low operating temperatures, i.e., 300–600 °C. Proton‐conducting candidates, in particular, hold a significant promise due to their low transport activation energy to deliver protons. Here, a unique hierarchical TiO_2_‐SrTiO_3_@TiO_2_ structure is developed inside an intercalated TiO_2_‐SrTiO_3_ core as “yolk” decorating densely packed flake TiO_2_ as shell, creating plentiful nano‐heterointerfaces with a continuous TiO_2_ and SrTiO_3_ “in‐house” interfaces, as well the interfaces between TiO_2_‐SrTiO_3_ yolk and TiO_2_ shell. It exhibits a reduced activation energy, down to 0.225 eV, and an unexpectedly high proton conductivity at low temperature, e.g., 0.084 S cm^−1^ at 550 °C, confirmed by experimentally H/D isotope method and proton‐filtrating membrane measurement. Raman mapping technique identifies the presence of hydrogenated HO─Sr bonds, providing further evidence for proton conduction. And its interfacial conduction is comparatively analyzed with a directly‐mixing TiO_2_‐SrTiO_3_ composite electrolyte. Consequently, a single fuel cell based on the TiO_2_‐SrTiO_3_@TiO_2_ heterogeneous electrolyte delivers a good peak power density of 799.7 mW cm^−2^ at 550 °C. These findings highlight a dexterous nano‐heterointerface design strategy of highly proton‐conductive electrolytes at reduced operating temperatures for SOFC technology.

## Graphical abstract

A unique hierarchical TiO_2_‐SrTiO_3_@TiO_2 _structure, inside an intercalated TiO_2_‐SrTiO_3 _core as “yolk” decorating densely packed flake TiO_2_ as shell, creates plentiful nano‐heterointerfaces with a continuous TiO_2 _and SrTiO_3 _“in‐house” interfaces, as well the interfaces between TiO_2_‐SrTiO_3 _yolk and TiO_2 _shell. It exhibits a low activation energy of 0.225 eV, and high proton conductivity as 0.084 S cm^−1^ at 550 °C.

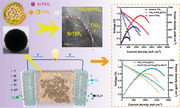

## 1 Introduction

Developing an alternative electrolyte enabling fast‐ion conduction for low‐temperature solid oxide fuel cells (LT‐SOFCs, particularly 300–600 °C) is challenging.^[^ ^1^ ^]^ Thereinto, proton‐conducting ceramics hold considerable attentions owing to low activation energy to leverage proton transfer.^[^ ^2^ ^]^ For a proton conductor, typically perovskite‐type BaZr_0.8_Y_0.2_O_3‐δ_ (BZY) and BaCe_0.7_Zr_0.1_Y_0.2_O_3‐δ_ (BCZY), oxygen vacancy in the crystal lattice is believed as the indispensable sites to assist the proton hopping and thus implement high protonic conduction.^[^ ^3^, ^4^ ^]^ However, the conductivity is still undesirable because of strongly restricted grain boundaries impedance, leading to an unpleasant high activation energy.^[^ ^5^ ^]^ Feasibly, a rational design for proton‐conducting electrolytes with flexible proton transport paths is likely a promising approach.

In response, considerable efforts have been devoted to designing potential proton‐conducting electrolytes.^2^, ^6^ ^]^ For example, Daniel et al. proposed a highly textured, epitaxially oriented BZY films deposited on a single‐crystalline (100)‐oriented MgO substrate via a pulsed laser technique, reducing grain boundary impedance and thus achieving a desirable proton conductivity as 0.11 S cm^−1^ at 500 °C.^[^ ^7^ ^]^ However, the implementation of this process necessitates intricate procedures and incurs substantial expenses, posing significant challenges for achieving widespread commercial viability. Garcia‐Barriocanal^[^ ^8^ ^]^ et al conducted a deep‐going study of the heterointerface of yttria‐stabilized zirconia (YSZ)/SrTiO_3_. Their findings reported that the partial occupancy and high disorder in the heterointerface oxygen plane, resulting in the introduction of a significant number of interfacial oxygen vacancies and a substantial decrease in the activation energy for ion migration, thereby increasing the ionic conductivity. Besides, enormous evidences relative to proton conduction give an alternative avenue through interfaces, such as in YSZ/ZnO,^[^ ^9^ ^]^ CeO_2_/CeO_2−δ_,^[^ ^10^ ^]^ SrTiO_3_/CeO_2_,^[^ ^11^ ^]^ and La/Pr co‐doped CeO_2_ (LCP)/ZnO.^[^ ^12^ ^]^ However, the high activation energy (0.49–0.62 eV) of ion transfer brings a big gap to implement desirable proton conductivity as SOFC electrolytes. Still, developing a highly conductive proton conductor with well‐tailored nano‐heterointerfaces to achieve desirable fuel cell performance is a significant challenge.

Herein, we develop a unique hierarchical TiO_2_‐SrTiO_3_@TiO_2_ structure with plentiful nano‐heterointerfaces inside intercalated TiO_2_‐SrTiO_3_ yolk/core decorating densely packed flake TiO_2_ particles as shell. A bicontinuous TiO_2_‐SrTiO_3_ interpenetrating network inside core can provide plentiful “in‐house” nano‐heterointerfaces, and further reinforce the interface paths via combing its TiO_2_ shell. In comparison with the conventional composite electrolytes obtained by a direct mixture approach, the special yolk–shell architecture establishes a more abundant and continuous network to provide a potential venue for proton transfer. The designed TiO_2_‐SrTiO_3_@TiO_2_ electrolyte exhibits an unexpectedly high proton conductivity and peak power density at low temperature. Additionally, a proton‐filtering membrane experiment and H/D isotopic effect via the electrochemical impedance spectroscopy (EIS) testing also were employed to verify the dominant proton conduction in the TiO_2_‐SrTiO_3_@TiO_2_ heterogeneous electrolyte. The implementation of this novel yolk–shell structure gives insights into designing proton‐conducting nano‐heterointerfaces for LT‐SOFC electrolytes.

## 2 Results

### 2.1 Structural Design of Spherical TiO_2_‐SrTiO_3_@TiO_2_ Yolk–Shell Heterostructure

**Figure 1:**
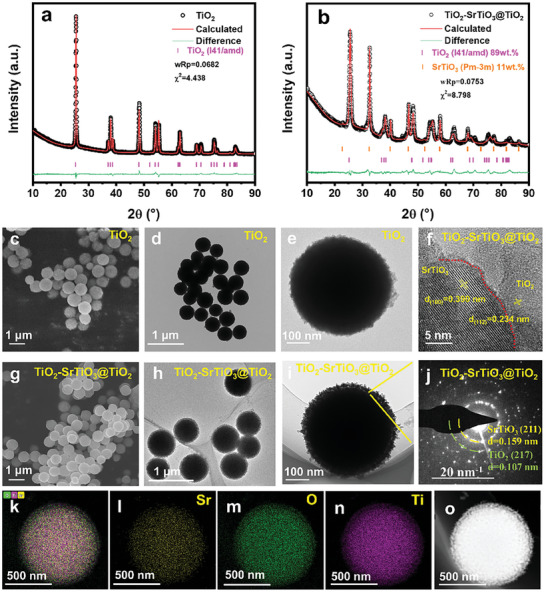
Structural characterizations. XRD patterns of a) TiO_2_, b) TiO_2_‐SrTiO_3_@TiO_2_; c) SEM and d,e) TEM diagrams of TiO_2_; g) SEM, h,i) TEM, f) HR‐TEM and corresponding SAED (j), and EDS images (k–n) of TiO_2_‐SrTiO_3_@TiO_2_. (o) HAADF‐STEM image of TiO_2_‐SrTiO_3_@TiO_2_.

The spherical TiO_2_ was successfully synthesized by a simple hydrolysis method. The Rietveld structure refinement on X‐ray powder diffraction (XRD), shown in **Figure** **1**a, revealed that the TiO_2_ (ICSD: 93 098) is anatase phase with I41/amd space group. The spherical morphology was confirmed by the scanning electron microscope (SEM) and transmission electron microscope (TEM) as shown in Figure 1c–e. Using spherical TiO_2_ as template, a yolk–shell structure with a bi‐continuous TiO_2_‐SrTiO_3_ interpenetrating structure core decorating with densely packed flake TiO_2_ shell was designed, namely as TiO_2_‐SrTiO_3_@TiO_2_. And its XRD pattern was refined by Rietveld method in Figure 1b. Clearly, an anatase TiO_2_ (ICSD: 93 098) and cubic SrTiO_3_ (ICSD: 186 708) perovskite were detected. The mass ratio of TiO_2_ to SrTiO_3_ is roughly calculated as 9:1 in TiO_2_‐SrTiO_3_@TiO_2_. Figure 1c–e,g–i shows the SEM and TEM images of bare TiO_2_ and TiO_2_‐SrTiO_3_@TiO_2_, demonstrating well‐dispersed spherical particles. Both the synthesized TiO_2_ and TiO_2_‐SrTiO_3_@TiO_2_ show similar size as approximately 500–600 nm, as shown in the Figure 1c,g. From the Figure 1d,h, TiO_2_‐SrTiO_3_@TiO_2_ has an obvious coating structure compared with TiO_2_. Figure 1i illustrates that the shell of TiO_2_‐SrTiO_3_@TiO_2_ consists of densely packed flake‐like particles.

The high‐resolution transmission electron microscopy (HR‐TEM) and corresponding selected area electron diffraction (SAED) are employed to detect the microstructure of TiO_2_‐SrTiO_3_@TiO_2_, shown in Figure 1f,j. From the Figure 1f, a clear interface between TiO_2_ and SrTiO_3_ was observed by HR‐TEM. The lattice spacing on the left side of the red dividing line is 0.390 nm, corresponding to the (100) plane of perovskite SrTiO_3_, and the lattice spacing on the right is 0.234 nm, corresponding to the (112) plane of anatase TiO_2_. The red dotted line is the heterogeneous interface formed between SrTiO_3_ and TiO_2_. The polycrystalline ring in SAED image in Figure 1j formed from the (211) plane of SrTiO_3_ and the (217) plane of TiO_2_, respectively, which also confirmed the interfacial structure of TiO_2_‐SrTiO_3_@TiO_2_. To further analyze the changes in the material before and after the interface construction, we employed the electron paramagnetic resonance (EPR) spectra to comparatively analyze oxygen vacancy concentrations of both TiO_2_ and TiO_2_‐SrTiO_3_@TiO_2_, displayed in Figure S1 (Supporting Information). Here, the presence of oxygen vacancies is reflected by the peak magnetic field intensity falling within the range of 3400 to 3600.^[^ ^13^ ^]^ A noticeable increase of peak intensity, implying an increase of oxygen vacancy concentration, was observed in TiO_2_‐SrTiO_3_@TiO_2_ compared with the bare TiO_2_.^[^ ^8^ ^]^ Figure S2c,f (Supporting Information) presents the X‐ray photoelectron spectra (XPS) of O 1s in TiO_2_ and TiO_2_‐SrTiO_3_@TiO_2_, respectively. As depicted, the surface O^2−^ concentration in the coated TiO_2_‐SrTiO_3_@TiO_2_ exhibits a substantial increase. This observation also suggests that the formation of heterogeneous interfaces benefits to create oxygen vacancies inside the TiO_2_‐SrTiO_3_@TiO_2_ sample. From the EDS mapping of Figure 1k–n, the Sr element is uniformly distributed within the spherical core, whereas the Ti and O elements can be detected in both the spherical shell and core. This yolk‐shell structure is clearly visible in the high‐angle annular dark field scanning transmission electron microscopy (HAADF‐STEM) image in Figure 1o, revealing a distinct yolk‐shell heterogeneous interface.

### 2.2 Electrochemical Performance and Proton Conduction of TiO_2_‐SrTiO_3_@TiO_2_

**Figure 2:**
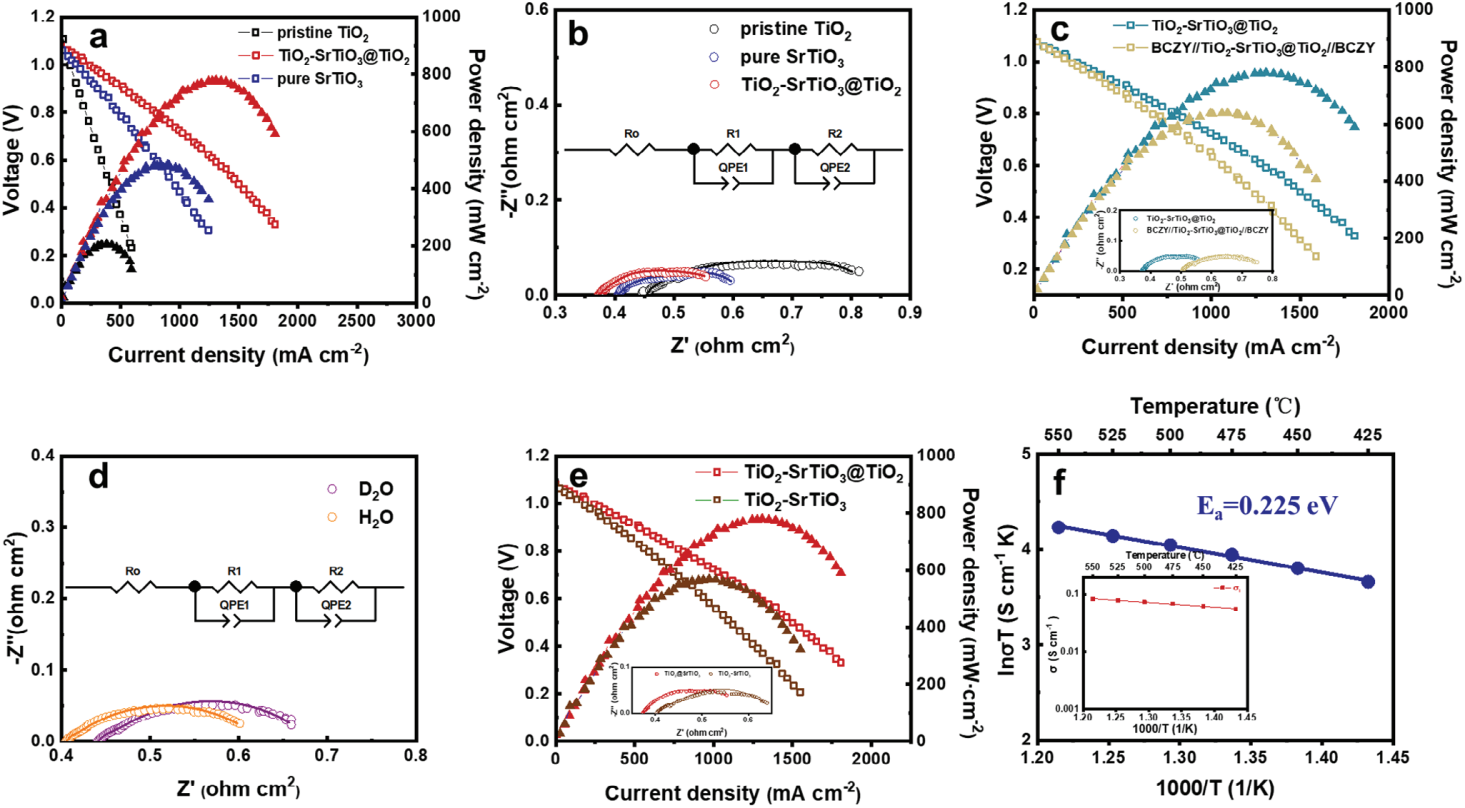
Validation for interfacial proton conduction. IP‐IV curves and EIS of a,b) TiO_2_, SrTiO_3_, TiO_2_‐SrTiO_3_@TiO_2_, and c) BCZY//TiO_2_‐SrTiO_3_@TiO_2_//BCZY electrolyte (the corresponding EIS curves are presented in inset image) at 550 °C. d) EIS using H_2_O, D_2_O at 550 °C. e) IP‐IV curves and corresponding EIS results (in inset image) of TiO_2_‐SrTiO_3_ and TiO_2_‐SrTiO_3_@TiO_2_ at 550 °C. f) Ionic conductivity (in inset image) and activation energy of TiO_2_‐SrTiO_3_@TiO_2_ as a function of 1000/T obtained from EIS and at 425–550 °C.

The current density‐voltage (*I*–*V*) and current density‐power density (I‐P) characteristics of the designed TiO_2_‐SrTiO_3_@TiO_2_ heterostructure electrolyte in a SOFC device using Ni_0.8_Co_0.15_Al_0.05_LiO_2‐δ_ coating on Ni form (NCAL‐Ni) as both anode/cathode measured at 550 °C are presented in **Figure** **2**a. All the open circuit voltages (OCVs) of the devices are above 1.0 V, indicating no short circuit or gas leakage during fuel cell running. Similarly, the SOFC pellets using the pristine TiO_2_ and SrTiO_3_ electrolytes were also respectively constructed. It only shows the PPD of 206.3 mW cm^−2^ and the OCV of 1.11 V, which is slightly lower than the reported result of the single fuel cell device using the TiO_2_ thin film electrolyte.^[^ ^14^ ^]^ while the PPD for the SrTiO_3_ electrolyte case is 482.8 mW cm^−2^ with an OCV of 1.06 V at 550 °C. This is due to the fact that neither TiO_2_ nor SrTiO_3_ are good ionic conductors for H^+^/O^2‐^.^[^ ^14^, ^15^ ^]^ Benefiting from the plentiful nano‐heterointerfaces, the PPD of the device using TiO_2_‐SrTiO_3_@TiO_2_ heterostructure electrolyte reaches up to 799.7 mW cm^−2^ at 550 °C, corresponding to an OCV of 1.08 V.

To further study the electrochemical mechanism, the electrochemical impedance spectroscopy (EIS) was carried out.^[^ ^16^ ^]^ As shown in Figure 2b, the intercept of the impedance arc on the real axis at high frequencies represents the ohmic resistance (R_o_), generally including the ionic transport resistance in the electrolyte and the electron migration resistance in the electrodes.^[^ ^17^ ^]^ And the NCAL‐Ni electrodes with excellent electronic conductivity contributes a negligible ohmic impedance. Polarization resistance (R_p_) is defined as the difference between the high‐frequency and low‐frequency intercept on the real axis of EIS plots, composed of several overlapping suppressed arcs.^[^ ^18^ ^]^ These suppressed arcs represent the physical and chemical processes corresponding to the hydrogen‐oxidation reaction (HOR) and oxygen‐reduction reaction (ORR) at the anode and cathode sides. As shown, the R_o_ and R_p_ for the TiO_2_‐SrTiO_3_@TiO_2_ pellet are 0.371 and 0.146 Ω cm^2^ respectively, which are much lower than the R_o_ of TiO_2_ (0.435 Ω cm^2^) and SrTiO_3_ (0.404 Ω cm^2^). It indicates that the designed structure of TiO_2_‐SrTiO_3_@TiO_2_ greatly accelerates the ionic conduction. The specific values are given in Table S1 (Supporting Information).

To ascertain the proportion of proton conduction in TiO_2_‐SrTiO_3_@TiO_2_, a diaphragm test utilizing BCZY was carried out.^[^ ^19^ ^]^ A fuel cell device was fabricated using a proton‐conducting BCZY filter, which involved the placement of a TiO_2_‐SrTiO_3_@TiO_2_ layer between two BCZY ion‐filter layers, effectively allowing only protons to traverse through and facilitating the evaluation of proton transport characteristics. As depicted in Figure 2c, the PPD of the fuel cell, that is Ni‐NCAL//BCZY//TiO_2_‐SrTiO_3_@TiO_2_//BCZY//NCAL‐Ni, reaches 639.1 mW cm^−2^ at 550 °C. This value accounts for ≈82% of the whole power output of the device without the BCZY membrane, thus confirming that protons serve as the primary transporting ions. In addition, it is important to note that the power loss cannot be solely attributed to the obstruction of oxygen ion transport. The data presented in the inset image for Figure 2c indicates that the resistance values, both the R_o_ and R_p_, for the pellet using BCZY filter, are higher compared to the ones without the filter. Specifically, the R_o_ with BCZY diaphragm is 0.490 Ω·cm^2^ and Rp is 0.293 Ω·cm^2^, while R_o_ and R_p_ are respectively as 0.404 and 0.211 Ω·cm^2^ for in the ones in absence of BCZY filter. This can be attributed to the increase in electrolyte thickness and the presence of two additional interfaces (BCZY and TiO_2_‐SrTiO_3_@TiO_2_), which also contribute to extra ohmic and polarization losses.

To further confirm the proton conduction in the TiO_2_‐SrTiO_3_@TiO_2_ heterogeneous electrolyte, we conducted an isotopic effect test using deuterium water (D_2_O).^[^ ^10^, ^20^ ^]^ If protons are indeed the dominant charge carriers, the H/D isotope would have a significant impact on its electrical conductivity. This isotopic effect arises from the disparity in ground state energies between O─H and O─D bonds. Since deuterium has a higher mass than hydrogen, it diffuses at a slower rate, leading to a discrepancy in electrical conductivity. By dissolving gaseous H_2_O and D_2_O in the electrolyte, H^+^ and D^+^ ions are introduced. The EIS results shown in Figure 2d and Table S2 (Supporting Information) clearly demonstrate that the R_o_ in the presence of H_2_O (0.399 Ω cm^2^) is lower compared to that in D_2_O (0.440 Ω cm^2^). This isotopic deduction experiment indicates a dominant proton conduction in the TiO_2_‐SrTiO_3_@TiO_2_. These findings further validate the test results depicted in Figure 2c.

To verify the superiority of the heterogeneous interface constructed by TiO_2_‐SrTiO_3_@TiO_2_, we compared it with a directly‐mechanically mixed TiO_2_‐SrTiO_3_ sample. TiO_2_‐SrTiO_3_ is obtained via mechanical grinding for 30 min in a mass ratio of TiO_2_ and SrTiO_3_ at 9:1. Figure 2e shows the IP‐IV and EIS between the TiO_2_‐SrTiO_3_ compared with that of the designed yolk–shell TiO_2_‐SrTiO_3_@TiO_2_. Considering the large size and irregular shape of SrTiO_3_ and TiO_2_ particles, achieving uniform mixing through mechanical methods would be difficult, the number of heterogeneous interfaces formed through directly mixing is significantly less than that of TiO_2_‐SrTiO_3_@TiO_2_. Expectedly, the PPD of the TiO_2_‐SrTiO_3_ is higher than pure TiO_2_ and SrTiO_3_, reaching 568.7 mW cm^−2^ at 550 °C. Therefore, we can conclude that the mechanical grinding can produce a certain number of heterogeneous interfaces, but far less than the designed interfaces of TiO_2_‐SrTiO_3_@TiO_2_ in aspects of the number and continuity. Based on the EIS diagram presented in the inset of Figure 2e, a significant disparity in the R_o_ between the two samples is evident. This observation indicates the importance of heterogeneous interfaces formed in aspect of enhancing ionic conduction.

Figure S3 (Supporting Information) shows the EIS results of TiO_2_‐SrTiO_3_@TiO_2_ measured at 425–550 °C with an interval of 25 °C. The slight changes of R_o_ prove the relatively low activation energy of proton transfer in TiO_2_‐SrTiO_3_@TiO_2_ electrolyte. We further calculated the ionic conductivity and activation energy and the results are shown in Figure 2f. The σ_i_ of TiO_2_‐SrTiO_3_@TiO_2_ can be obtained from the simulated ohmic resistance and calculated according to the equation: σ = L/(RS), where L and S stand for material thickness and area, respectively. As shown in the inset image of Figure 2f, the resultant ionic conductivity of TiO_2_‐SrTiO_3_@TiO_2_ was found to be 0.084 S cm^−1^ at 550 °C and 0.056 S cm^−1^ at 425 °C, which is significantly higher than that of BZY (0.01 S cm^−1^ at 600 °C).^[^ ^7^ ^]^ Furthermore, Figure 2f shows that the proton transfer activation energy in TiO_2_‐SrTiO_3_@TiO_2_ was found to be remarkably low to 0.225 eV, which is significantly less than the reported value of BZY as 0.48 eV.^[^ ^7^ ^]^ Although a thin Li‐doped TiO_2_ film electrolyte was proposed to transfer proton and the mixed ion conductivity of SrTiO_3_ electrolyte was attributed the emerging SrTiO_3_/Li_2_CO_3_ interfaces, the observed dominant proton conductivity in SrTiO_3_/TiO_2_ could infer the critical role of SrTiO_3_/TiO_2_ interfaces.^[^ ^14^, ^15^ ^]^ Therefore, we can draw an important conclusion that the formation of the heterogeneous interfaces enables proton conduction in the TiO_2_‐SrTiO_3_@TiO_2_.

### 2.3 Structural Evolution of TiO_2_‐SrTiO_3_@TiO_2_ Heterogeneous Electrolyte

**Figure 3:**
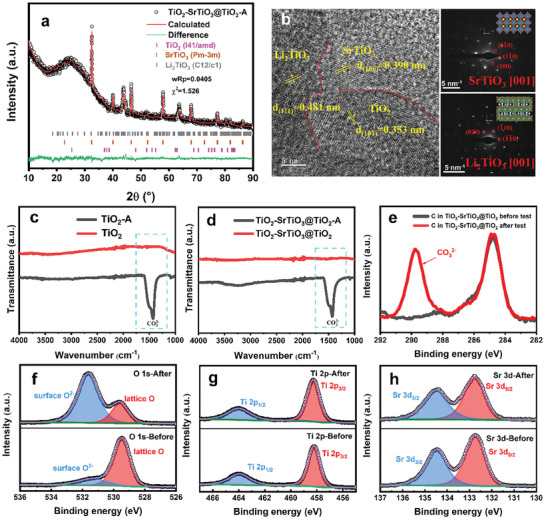
Structural evolution. a) XRD and b) HR‐TEM and SAED images of TiO_2_‐SrTiO_3_@TiO_2_‐A. c,d) FT‐IR spectra of TiO_2_ and TiO_2_‐SrTiO_3_@TiO_2_ before and after fuel cell operation. e–h) XPS fitting results of C 1s, O 1s, Ti 2p, and Sr 3d for the electrolyte before and after fuel cell operation.

The possible structure changes of the TiO_2_‐SrTiO_3_@TiO_2_ electrolyte during fuel cell operation are considered in this study. Consequently, an extensive set of characterization studies in **Figure** **3** was conducted to examine the structural evolution of the TiO_2_‐SrTiO_3_@TiO_2_ electrolyte after operation (denoted as TiO_2_‐SrTiO_3_@TiO_2_‐A) in details. In Figure 3a, we found that TiO_2_ was partially transformed into Li_2_TiO_3_ (ICSD: 1 515 995) after suffering fuel cell operation. And Li_2_CO_3_ are detected, which might be generated owing to the reduction of NCAL anode in H_2_, and then flow to the electrolyte.^[^ ^21^ ^]^ Figure 3b is the HR‐TEM and SAED images of the TiO_2_‐SrTiO_3_@TiO_2_‐A. In addition to TiO_2_ and SrTiO_3_, the lattice of Li_2_TiO_3_ can be also clearly seen here, might be caused by the migration of Li^+^ from the NCAL electrode to the electrolyte.^[^ ^22^ ^]^

The Fourier transform infra‐red spectroscopy (FT‐IR) analysis on TiO_2_ and TiO_2_‐SrTiO_3_@TiO_2_ electrolytes further explores the alteration during fuel cell testing, as presented in Figure 3c,d. It shows that Li_2_CO_3_ can be found in both tested TiO_2_ and TiO_2_‐SrTiO_3_@TiO_2_ electrolyte. The cross‐sectional SEM and EDS mapping of the fuel cell before and after operation are depicted in Figures S4 and S5 (Supporting Information), respectively. It exhibits a distinct sandwich structure, showing that Ni and Co are distributed in both electrodes, Sr and Ti elements are mainly in the middle electrolyte layer, and O element is observed in both the electrode and electrolyte layers. Figure S4a (Supporting Information) displays the SEM image and a magnified view of the electrolyte before operation, revealing spherical particles with discernible gaps between them. Subsequently, the SEM image for the electrolyte after suffering fuel cell operation, and corresponding an enlarged view are depicted in Figure S5a (Supporting Information), illustrating the emerging amorphous Li_2_CO_3_ with relatively dense microstructure. This phenomenon occurs due to the capillary action‐driven diffusion of Li_2_CO_3_ from the electrode to the electrolyte during fuel cell operation. And this process serves to improve the gas tightness of the electrolyte layer, and simultaneously improve the ion conduction to a certain extent.^[^ ^23^ ^]^ However, according to the results of IV‐IP curves in Figure 2a,e), the performance of each pellet still with a huge gap. The device using TiO_2_‐SrTiO_3_ demonstrates a higher peak power density compared to pure TiO_2_ and SrTiO_3_. Moreover, the abundance of SrTiO_3_/TiO_2_ heterointerfaces in TiO_2_‐SrTiO_3_@TiO_2_ led to a remarkable enhancement in the fuel cell performance, reaching up to 799.7 mW cm^−2^. This further shows that the key factor to enhance the performance is the heterogeneous interface formed in the TiO_2_‐SrTiO_3_@TiO_2_. Figure 3e,f presents the XPS results of the C and O 1s in TiO_2_‐SrTiO_3_@TiO_2_ before and after testing, respectively. In the tested TiO_2_‐SrTiO_3_@TiO_2_, a significant presence of CO_3_^2−^ is observed. Additionally, Figure 3g,h compares the XPS results of Ti 2p and Sr 3d, respectively, showing no noticeable change and indicating the stability of the valence state of both Ti and Sr within TiO_2_‐SrTiO_3_@TiO_2_ during testing.

### 2.4 Proton Conduction

**Figure 4:**
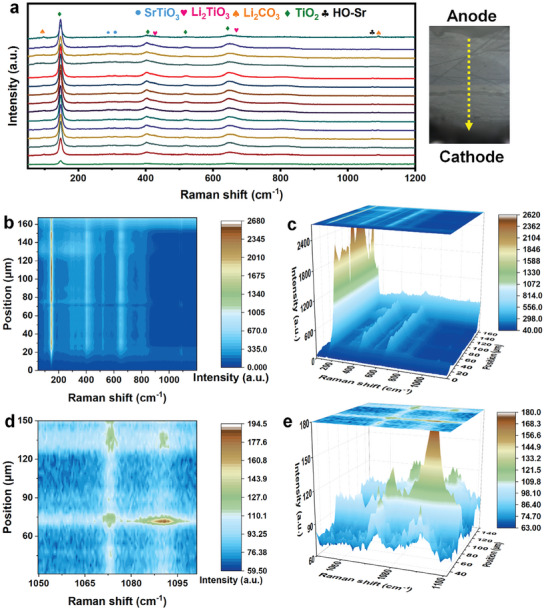
a) Raman mapping of the TiO_2_‐SrTiO_3_@TiO_2_ electrolyte layer from anode to cathode. (b) 2D and c) 3D images of full range, and the d) 2D and e) 3D images at 1050–1100 cm^−1^ of the spectrum.

The electrolyte after operation was treated by cross section polisher (CP) and then tested by Raman mapping, and the points were taken from anode to cathode. The peak positions of various substances are illustrated in **Figure** **4**a. The peaks observed at 97 and 1090 cm^−1^ can be attributed to Li_2_CO_3_. The Raman peaks at 147, 403, 519, and 647 cm^−1^ correspond to TiO_2_, while the characteristic peaks of Li_2_TiO_3_.^[^ ^24^ ^]^located at 430 and 668 cm^−1^, and the peaks of 290 and 326 cm^−1^ belong to SrTiO_3_.^[^ ^25^ ^]^ Moving on to Figure 4b,c, these images depict the 2D and 3D representations of the full‐spectrum Raman mapping of the tested electrolyte, respectively. As shown, the Raman characteristic peak of TiO_2_ on both sides of the electrolyte significantly decreases after operation. Conversely, the peak of Li_2_TiO_3_ at this position shows a noticeable increase, indicating the Li^+^ in the electrode migrates and becomes embedded in TiO_2_, resulting in the transformation of a portion of TiO_2_ into Li_2_TiO_3_. This observation is consistent with the XRD results depicted in Figure 3a.

Figure 4d,e displays the 2D and 3D Raman spectra, respectively in the range of 500–600 cm^−1^ and 1050–1100 cm^−1^. The presence of a characteristic peak at 1071 cm^−1^ confirms the formation of HO‐Sr, providing direct evidence of the proton transport in the electrolyte.^[^ ^26^ ^]^ Compared with the SrTiO_3_, TiO_2_, and TiO_2_‐SrTiO_3_@TiO_2_ powder samples before operation in Figure S6 (Supporting Information), this peak corresponds to the new bond formed by the absorption of protons by SrTiO_3_. Our experimental results demonstrate that the enhanced protonic conduction observed in TiO_2_‐SrTiO_3_@TiO_2_ is concerned with the presence of more active Sr─O at the heterointerface, which could potentially be produced along with the generation of oxygen vacancies.

## 3 Discussion

The TiO_2_‐SrTiO_3_@TiO_2_ exhibits a higher ionic conductivity of 0.084 S cm^−1^ and a lower activation energy of proton transfer at 0.225 eV at 550 °C, in comparison to the typically proton conductor BZY (0.01 S cm^−1^ at 600 °C, activation energy of 0.48 eV),^[^ ^7^ ^]^ thus demonstrating a desirable PPD of 799.7 mW cm^−2^ at 550 °C. The ultra‐low activation energy for proton transfer indicates a distinct transport path, i.e., heterogeneous interface, comparable with the traditional protonic conductor via bulk oxygen vacancy. The high interfacial ionic conduction arisen of TiO_2_‐SrTiO_3_@TiO_2_ is mainly due to its unique bicontinuous TiO_2_‐SrTiO_3_ interpenetrating network as “yolk” combing a heterogeneous shell structure, facilitating the creation of numerous continuous protonic conduction paths. Furthermore, the TiO_2_/SrTiO_3_ heterogeneous interfaces result in the formation of oxygen vacancies, and enhancing the activity of lattice oxygen.^[^ ^8^, ^27^ ^]^ Through the decomposition of H_2_O and the catalysis of H_2_, oxygen vacancies (or active lattice oxygen) at the heterogeneous interface can combine with HO· (or H·) to realizing the macroscopic transport of H^+^,^[^ ^28^ ^]^ which may be the key to the proton transport at the heterogeneous interface. This is also further supported by the vibration peak of the HO‐Sr bond at the 1071 cm^−1^ in the Raman mapping results. The designed TiO_2_‐SrTiO_3_@TiO_2_ relies on interfacial proton conduction skillfully and thus avoids the limitation of grain boundary impedance in typically proton conductor, bringing in a higher ionic conductivity and lower activation energy.

**Figure 5:**
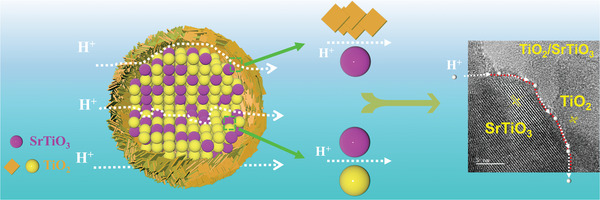
Schematic diagram. Proton conduction along the nano‐heterointerfaces consisting of a continuous TiO_2_ and SrTiO_3_ “in‐house” interfaces and the interfaces between TiO_2_‐SrTiO_3_ yolk and TiO_2_ shell.

Based on this analysis, a schematic diagram of proton interfacial transport in TiO_2_‐SrTiO_3_@TiO_2_ electrolytes was given in **Figure** **5**. Finally, the fact the results of experiments and Raman mapping exhibit conclusion strengthens our argument that proton transfer occurs at heterointerface by oxygen vacancies and active lattice oxygen. This provides a new strategy to design high performance electrolytes, that is, based on heterogeneous interface characteristics to explore and develop advanced proton‐conducting electrolytes.

In conclusion, we have successfully designed and fabricated a unique yolk‐shell TiO_2_‐SrTiO_3_@TiO_2_ electrolyte with abundant heterogeneous interfaces via a facile hydrothermal method. As SOFC electrolyte, it achieved an impressive OCV of 1.08 V and a PPD of 799.7 mW cm^−2^ at 550 °C. The remarkable results can be attributed to the presence of numerous heterogeneous interfaces. Through a multitude of experiments, it has been demonstrated that protons serve as the primary transport ions in TiO_2_‐SrTiO_3_@TiO_2_, demonstrating movement along the heterogeneous interfaces. At the heterogeneous interface of TiO_2_‐SrTiO_3_@TiO_2_, the H^+^ ions combine with the Sr─O bond and then forms a reactive HO‐Sr bond to facilitate proton transfer. This phenomenon highlights the crucial role of the heterogeneous interface in facilitating the proton conduction process. More broadly, this work provides a new idea and method for designing and exploiting novel proton‐conducting electrolyte candidates for LT‐SOFCs.

## 4 Experimental Section

### Preparation of Spherical TiO_2_

The 200 mL aqueous solution of Ti(SO_4_)_2_ (3.84 g) was mixed with the 1‐propanol (300 ml) solution of PVP (8.0 g) to form a suspension. The mixture was stirred for 3 h at 70 °C and then aged in the 1 mol L^−1^ KOH solution for 15 h. The final amorphous spherical TiO_2_ powder was obtained after washing repeatedly with water and alcohol and then dried at 60 °C for 12 h.

### Preparation of TiO_2_‐SrTiO_3_@TiO_2_

0.5 mmol TiO_2_ was added to 40 mL deionized water to form A suspension, 0.5 mmol Sr(NO_3_)_2_ was dissolved in 120 mL to form B solution, A was poured into B solution under intense stirring, and continuously for 30 min to make it uniformly dispersed. Subsequently, the suspension was poured into 200 mL Teflon reactor and suffering a hydrothermal reaction at 180 °C for 3 h. The sample was collected through a high‐speed centrifuge and then washed with deionized water followed by ethanol; the dried powder was obtained at 60 °C for 12 h in the oven. The TiO_2_‐SrTiO_3_@TiO_2_ heterostructures were obtained with post heat‐treatment at 800 °C for 3 h with a heating rate of 3 °C min^−1^.

### Fuel Cell Fabrication

SOFCs devices based on the various electrolytes were fabricated via a dry pressing method. A semiconductor Ni_0.8_Co_0.15_Al_0.05_LiO_2‐δ_ (NCAL, Tianjin Bamo Technology Co., Ltd., China.) was used as a symmetrical electrode in the form of NCAL‐pasted Ni‐foam (NCAL‐Ni), in which the PPI of Ni‐foam is 60–110. The NCAL‐Ni electrodes were prepared by blending NCAL powders with terpineol solvent to create a slurry. This slurry was subsequently applied onto Ni‐foam and dried at 120 °C for 15 min, resulting in the formation of NCAL‐Ni components. The Ni‐foam was used to guarantee the mechanical strength of the cell and sustain the porous structure of the electrode.

Following a typical fuel cell fabrication procedure, the various TiO_2_, SrTiO_3_ (Beijing Mreda Technology Co., Ltd.) and TiO_2_‐SrTiO_3_@TiO_2_ powder was compacted between two pieces of NCAL‐Ni electrodes uniaxially under a pressure load of 500 MPa into one pellet. The cell pellets with various electrolytes powders were assembled in a same configuration of NCAL‐Ni//TiO_2_‐SrTiO_3_@TiO_2_//NCAL‐Ni, with thicknesses of ≈1 mm and 0.64 cm^2^ in the active area. The thickness of electrolyte was ≈280 µm. All of these fuel cells underwent online preheating at 550 °C for 30 min before being operationalized and their performance measured.

### Material Characterizations and Electrochemical Measurements

The crystal structures of the TiO_2_ and TiO_2_‐SrTiO_3_@TiO_2_ bulk‐heterostructures were analyzed by D‐max‐2500/PC. The diffraction patterns were recorded in the 2θ range of 10°−90°. The microstructures of these specimens were investigated using a transmission electron microscope (TEM, HT7700) and a scanning electron microscope (SEM, supra‐55). Further interface investigations were performed using a Talos microscope. Raman spectroscopies were acquired by Raman spectrometer (Lab RAM Odyssey, Horiba Scientific) excited by 532 nm laser. The X‐ray photoelectron spectra (XPS) analysis was performed using a Thermo Scienti fic ESCALAB 250Xi spectrometer using an Al K α X‐ray source. The C 1s peak located at 284.8 eV was used to calibrate binding energy positions. The oxygen vacancy conditions of these specimens were investigated using an electron paramagnetic resonance (EPR, EMXPlus).

The electrochemical properties of all various electrolytes were performed on a CHI660e electrochemical workstation under OCVs mode of the cells by applying an AC voltage with amplitude of 0.1 V and frequency of 0.05–10^6^ Hz on the basis of the OCV. The current density‐voltage characteristics all various electrolytes fuel cell were measured using an IT8511 electronic load (ITECH Electrical Co., Ltd., China), and IT8500 software was used to record the data and modulate the scan speed in the current‐voltage sweep. The fuel cells were operated in the temperature at 550 °C with dry hydrogen and air as fuel and oxidant (100–150 mL min^−1^), respectively.

## Conflict of Interest

The authors declare no conflict of interest.

## Supporting information

Supporting Information

## Data Availability

The data that support the findings of this study are available from the corresponding author upon reasonable request.
