# Supplementary material for: Enriching Nano‐Heterointerfaces in Proton Conducting TiO2‐SrTiO3@TiO2 Yolk–Shell Electrolyte for Low‐Temperature Solid Oxide Fuel Cells
Source: Adv Sci (Weinh). 2024 Jun 12;11(36):2401008. doi: 10.1002/advs.202401008 (PMC11423155; doi:10.1002/advs.202401008)
Supplement: Supplementary file 1 — Supporting Information [file ADVS-11-2401008-s001.docx]

Supporting Information

**Enriching nano-heterointerfaces in proton conducting TiO_2_-SrTiO_3_@TiO_2_ yolk-shell electrolyte for low-temperature solid oxide fuel cells**

**Mengchen Du^1^, Shaozheng Ji^2,^ *, Pan Zhang^1^, Yongfu Tang^1,^ *, Yanyan Liu^1,^ ***

^1^State Key Laboratory of Metastable Materials Science and Technology (MMST), Hebei Key Laboratory of Applied Chemistry, Yanshan University, Qinhuangdao 066004, P.R. China

^2^Ultrafast Electron Microscopy Laboratory, School of Physics, Nankai University, Tianjin 300071, China

Corresponding authors: Y.Y. Liu ([liuyy@ysu.edu.cn](mailto:liuyy@ysu.edu.cn)); Y.F. Tang (tang[yongfu@ysu.edu.cn](mailto:yongfutang@ysu.edu.cn)) & S.Z. Ji ([jshaoz@nankai.edu.cn](mailto:jshaoz@nankai.edu.cn))


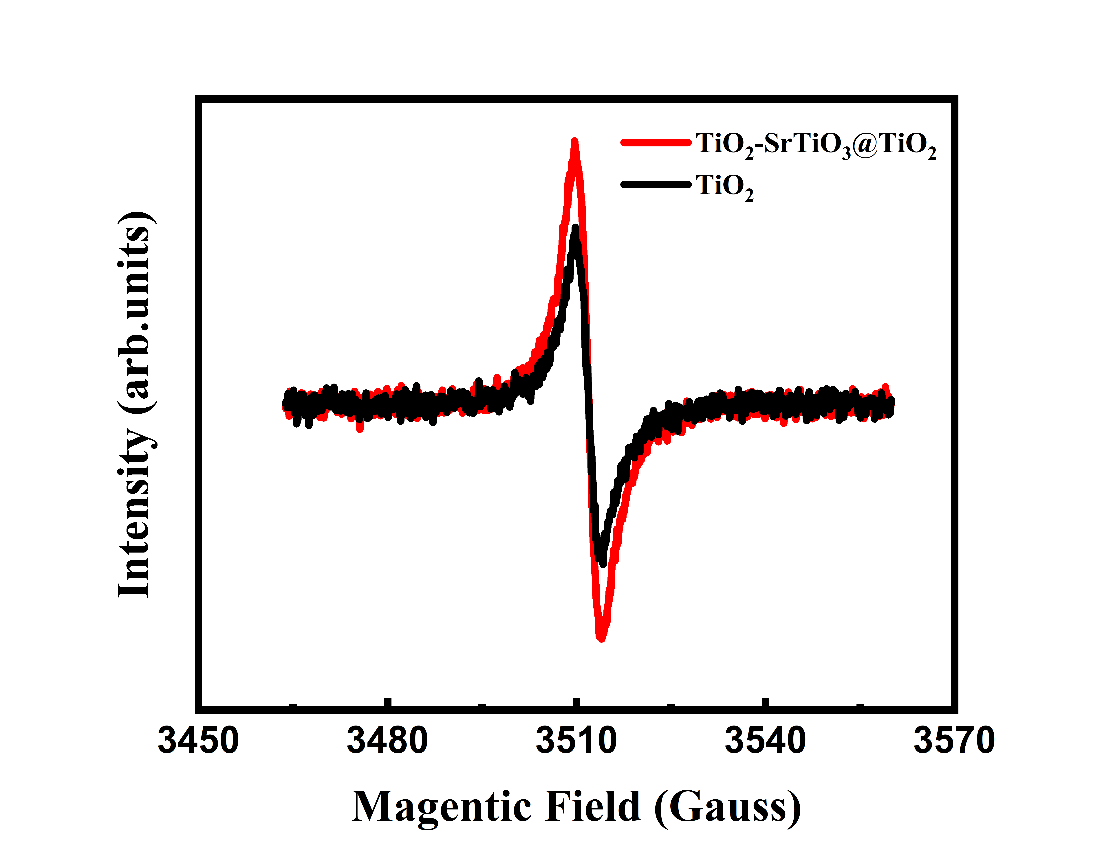
 **Fig. S1** EPR result of TiO_2_ and TiO_2_-SrTiO_3_@TiO_2_


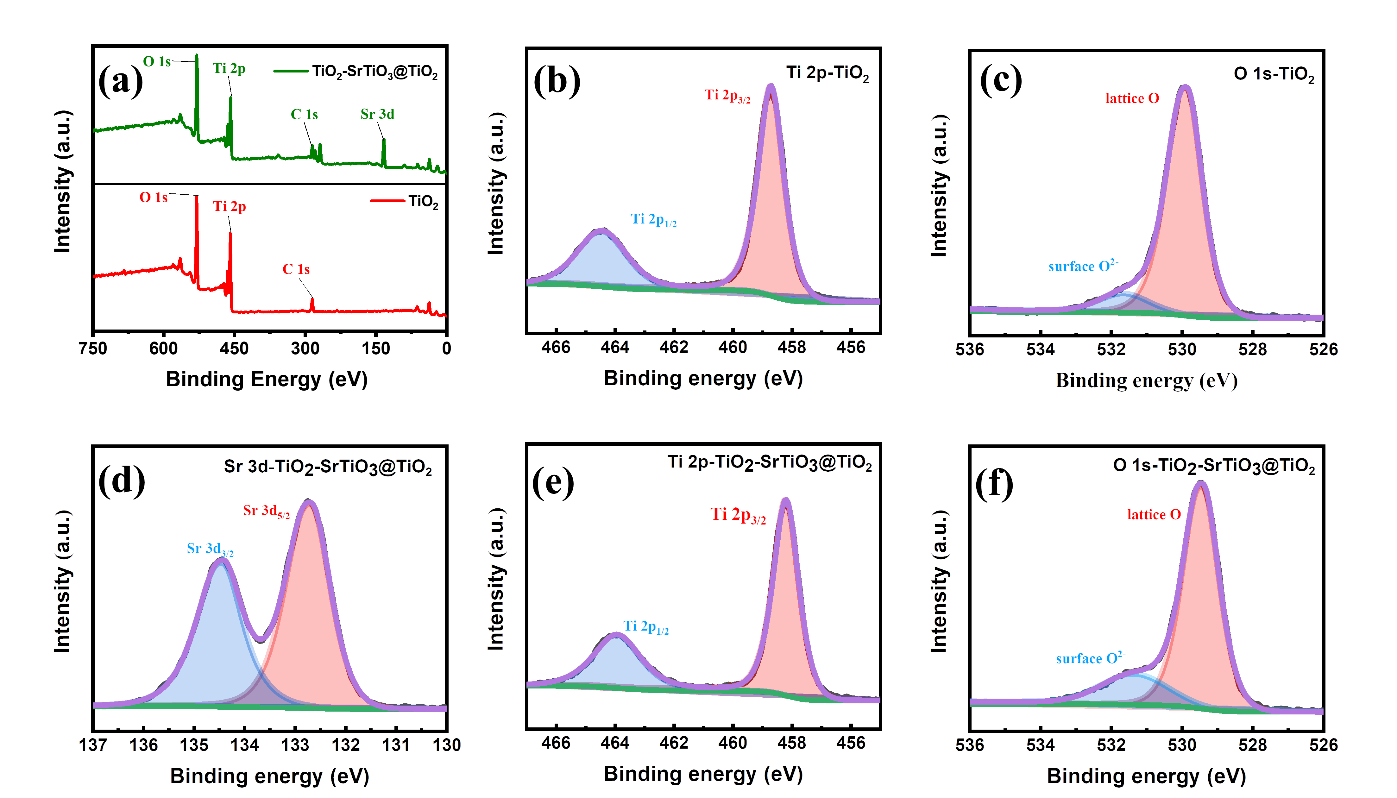


**Fig. S2** XPS results for the TiO_2_, and TiO_2_-SrTiO_3_@TiO_2_: (a)survey spectra, Ti 2p (b) and O 1s (c) in TiO_2_, Sr 3d (d), Ti 2p (e) and O1s (f) in TiO_2_-SrTiO_3_@TiO_2_

**
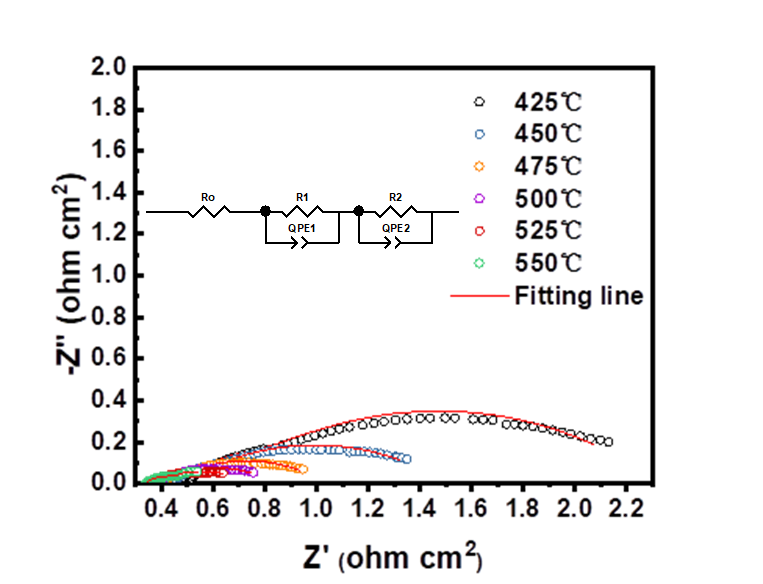
Fig. S3** EIS results of TiO_2_-SrTiO_3_@TiO_2_ at the temperature range of 425-550 ^o^C with an interval of 25 ^o^C


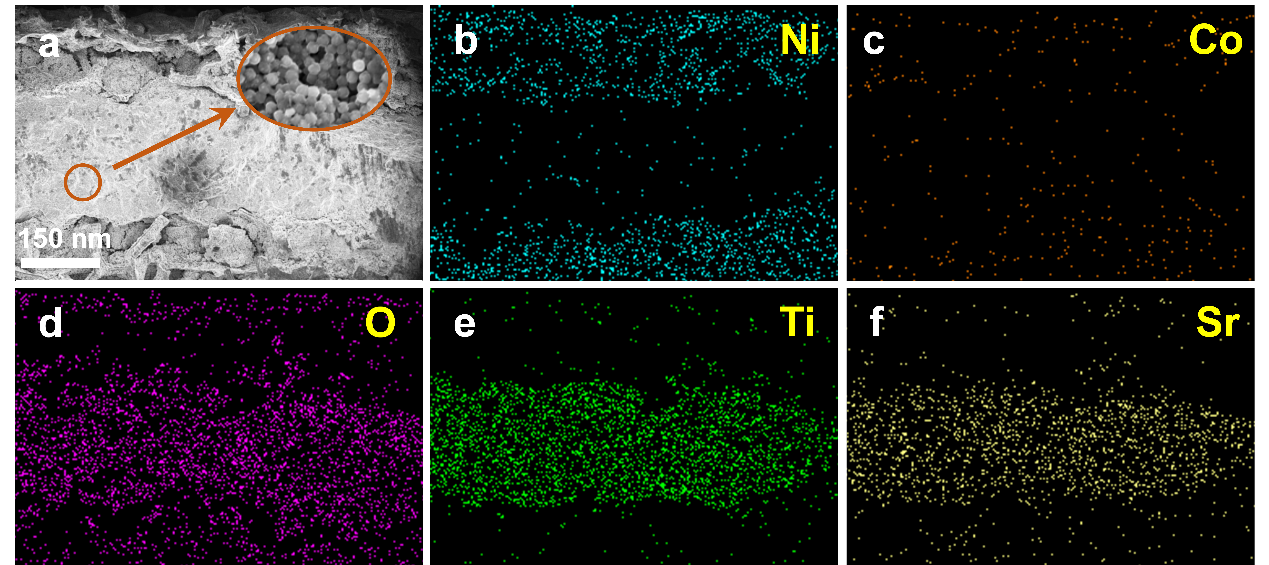


**Fig. S4** Cross-sectional fuel cell pellet: SEM image (a) and elemental mapping (b-f) before operation

**
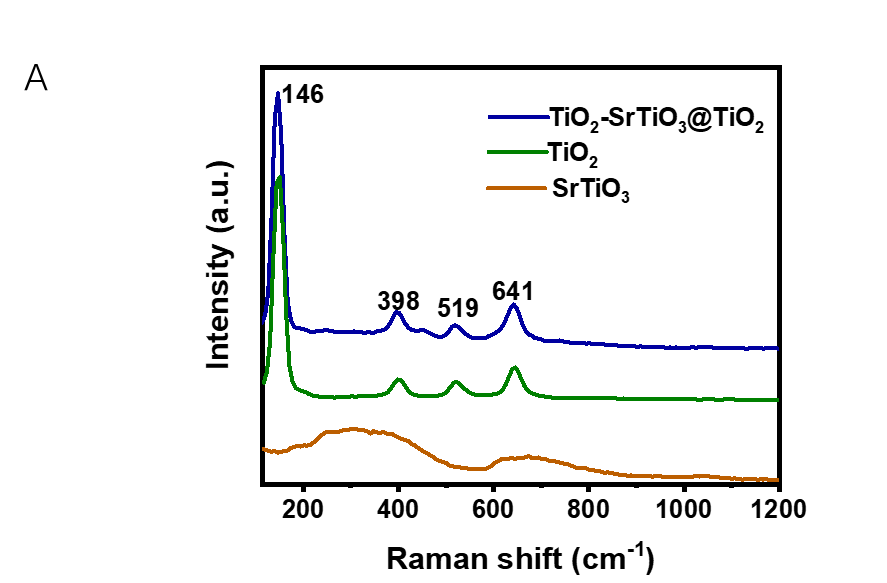
**
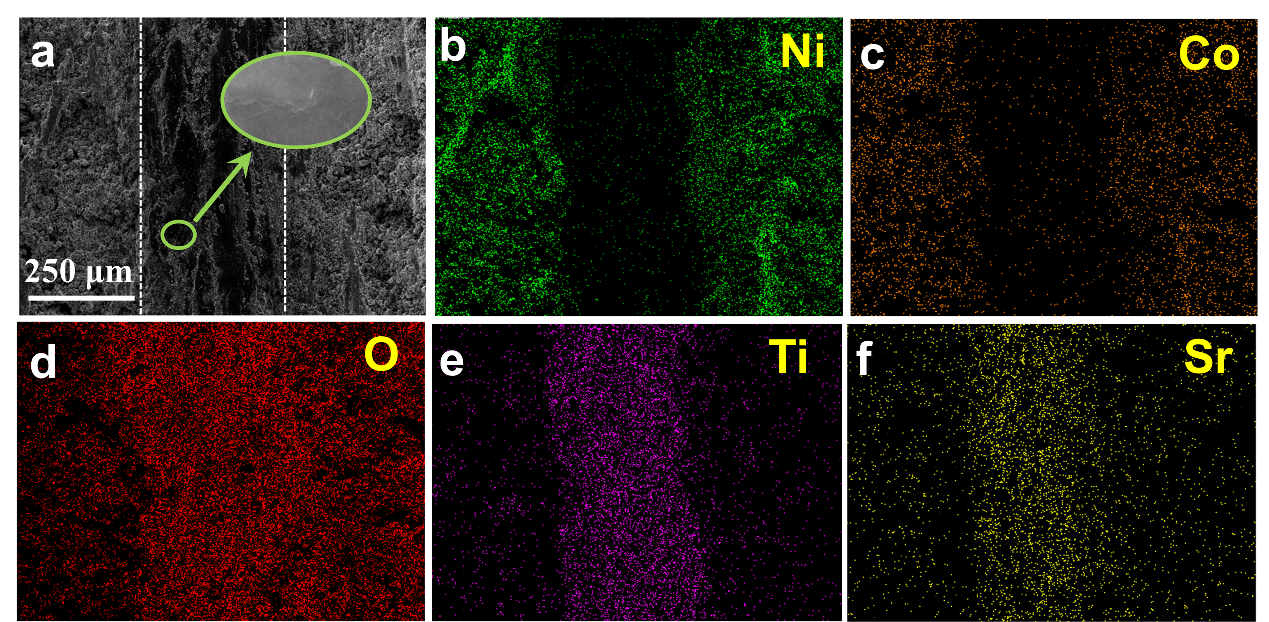
**Fig. S5** Cross-sectional pellet: SEM image (a) and elemental mapping (b-f) after suffering fuel cell measurement

**Fig. S6** Raman spectrum of the bare TiO_2_, SrTiO_3_, and TiO_2_-SrTiO_3_@TiO_2_ before fuel cell operation

**Table S1** The fitting results of impedance spectra in H_2_/air condition using the Ro(R_1_QPE_1_) (R_2_QPE_2_) circuit. Resistances are given in ohm cm^2^.

| Samples | Ro (ohm cm^2^) | R_1_ (ohm cm^2^) | R_2_ (ohm cm^2^) |
| --- | --- | --- | --- |
| TiO_2_ | 0.435 | 0.034 | 0.395 |
| SrTiO_3_ | 0.404 | 0.088 | 0.123 |
| TiO_2_-SrTiO_3_@TiO_2_ | 0.371 | 0.024 | 0.122 |
| TiO_2_-SrTiO_3_ | 0.404 | 0.033 | 0.235 |
| BCZY//TiO_2_-SrTiO_3_@TiO_2_//BCZY | 0.49 | 0.027 | 0.266 |

**Table S2** The fitting results of impedance spectra in different condition using the Ro(R_1_QPE_1_) (R_2_QPE_2_) circuit. Resistances are given in ohm cm^2^

| Atmosphere | Ro (ohm cm^2^) | R_1_ (ohm cm^2^) | R_2_ (ohm cm^2^) |
| --- | --- | --- | --- |
| H_2_O | 0.399 | 0.009 | 0.217 |
| D_2_O | 0.440 | 0.012 | 0.232 |

**Table S3** The fitting results of impedance spectra in different temperature using the Ro(R_1_QPE_1_) (R_2_QPE_2_) circuit. Resistances are given in ohm cm^2^

| Temperature (^o^C) | Ro (ohm cm^2^) | R_1_ (ohm cm^2^) | R_2_ (ohm cm^2^) |
| --- | --- | --- | --- |
| 550 | 0.334 | 0.101 | 0.016 |
| 525 | 0.351 | 0.049 | 0.285 |
| 500 | 0.365 | 0.451 | 0.019 |
| 475 | 0.398 | 0.610 | 0.025 |
| 450 | 0.439 | 0.996 | 0.061 |
| 425 | 0.500 | 1.667 | 0.015 |
